# 217 The Irish Sports Monitor 2011-2023: Data Driven Insights - The Story So Far

**DOI:** 10.1093/eurpub/ckae114.025

**Published:** 2024-09-26

**Authors:** Eric Lacey, Benny Cullen

**Affiliations:** Sport Ireland, Dublin, Ireland; Sport Ireland, Dublin, Ireland

## Abstract

First published in 2007 the Irish Sports Monitor (ISM) is a long-established survey providing an ongoing measurement of sports participation in Ireland. Led by Benny Cullen, director of Research and Innovation at Sport Ireland (SI), the ISM examines both recreational and competitive sport, as defined in the National Sports Policy (2018-2027). The ISM survey’s over 8,000 respondents annually, providing an important monitoring instrument for governmental policy makers while also acting as a critical reference point for many within the academic field of sports and social science in Ireland.

While the study focuses primarily on active sports participation it also provides important insights on club membership, volunteering, event attendance as well as broader physical activity including recreational walking, walking for transport and cycling for transport. More recently Sport Ireland researchers have embarked upon an ambitious data harmonisation journey using input from the ISM and other SI publications, combining over a decade of research datasets into one large ‘data lake’. This new development has enhanced the analytical capability of the organisation allowing for improved cross sectional time series analysis of the datasets. This new approach has helped uncover new insights into how sport and physical activity behaviours change and evolve in a population over time. Sport Ireland have also integrated census data and population projections from Ireland’s central statistics office to the ‘data lake’, which is being used to develop models of the future sport and physical activity behaviours and participation rates in Ireland, out to 2050. The insights from Sport Ireland’s research and the recently developed data lake are being used to assist in the design and development of innovative sport and physical activity programmes, identify and target marginalised communities and inform local and national policy makers on the emergent trends in the Irish sporting landscape. This presentation will demonstrate how a data guided tracking of changes in population dynamics and demographics can help us project and model future levels of sport and physical activity related behaviours. The potential for the development of an integrated multi-national database, built on a harmonised approach to data collection, will also be discussed.

